# Metagenomic Sequences of Three Drinking Water and Two Shower Hose Biofilm Samples Treated with or without Copper-Silver Ionization

**DOI:** 10.1128/MRA.01220-19

**Published:** 2020-01-16

**Authors:** Anke Stüken, Thomas H. A. Haverkamp

**Affiliations:** a Department of Zoonotic, Food- and Waterborne Infections, Norwegian Institute of Public Health, Oslo, Norway; b Department of Epidemiology, Norwegian Veterinary Institute, Oslo, Norway; Indiana University, Bloomington

## Abstract

We announce five shotgun metagenomics data sets from two Norwegian premise plumbing systems. The samples were shotgun sequenced on two lanes of an Illumina HiSeq 3000 instrument (THRUplex chemistry, 151 bp, paired-end reads), providing an extensive resource for sequence analyses of tap water and biofilm microbial communities.

## ANNOUNCEMENT

Water disinfection efficiently reduces the total number of bacteria in drinking water but may also select for disinfection-resistant communities (1). Several common water disinfection methods (2–6) and water flow through metal pipes (7) have been reported to increase the relative abundance of antibiotic-resistant bacteria (ARBs) and genes (ARGs) in drinking water systems.

The rationale for this pilot study was to determine the amount of sequencing required to detect and characterize ARGs in Norwegian premise plumbing systems and to investigate the impact of silver-copper ionization (CSI) on the number and type of antibiotic, biocide, and metal resistance genes detected. CSI is an in-house water disinfection method that works by releasing positively charged silver and copper ions directly into the water stream (8).

We announce three drinking water and two shower hose biofilm metagenomes. Samples were taken from two neighboring buildings in Oslo, Norway, both receiving water from the same drinking water treatment plant and through the same distribution pipes. One building used a CSI system as an additional water disinfection step; the other building did not.

Sampling and DNA isolation protocols are described in reference 9. Previous 16S rRNA gene analyses of the study system revealed five distinct bacterial community clusters (9). DNA from samples within each cluster were pooled in equal amounts prior to library synthesis to produce the metagenome samples described here. Libraries were created using Illumina THRUplex chemistry and were sequenced on two lanes of an Illumina HiSeq 3000 instrument (151 bp, paired-end reads [April 2017]).

**FIG 1:**
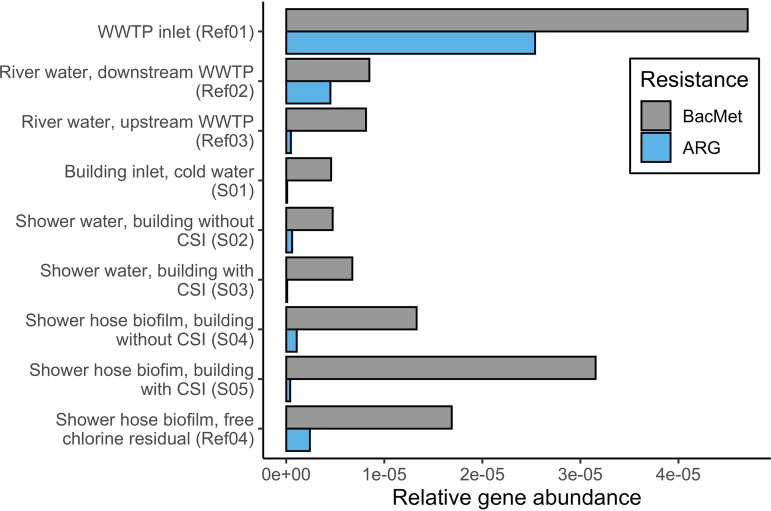
Relative abundance of antibacterial biocide and metal resistance (BacMet) genes and antibacterial resistance genes (ARGs) in the five metagenomes announced here (S01 to S05) and in four reference data sets. CSI, copper-silver ionization; WWTP, wastewater treatment plant.

To evaluate the results in relation to other human-influenced aquatic habitats known to contain resistance genes, we included the following four published metagenomic data sets in the analyses (Fig. 1): Ref01, inlet of a wastewater treatment plant (WWTP) (ENA accession number ERR1414237) (10); Ref02, river water upstream of a WWTP (SRR5306407); Ref03, river water downstream of a WWTP (SRR5298537) (11); and Ref04, hospital shower hose biofilm from a plumbing system with free chlorine (SRR2751194) (12). Reference data sets were quality treated and analyzed the same way in which the data sets presented here were.

Low-quality bases, reads, and sequencing adapters were trimmed with Trimmomatic v.036 (default settings, paired-end mode) (13). Exact duplicate reads (–derep: 14, –derep_min: 2) and low-complexity sequences (–lc_method: entropy, –lc_threshold cutoff: 70) were removed with Prinseq v.0.20.4 (14). The data sets were screened for coliphage phiX (GenBank accession number NC_001422.1) and human sequences (hg19) with BBMap v.37.53 (default settings) (15). Unpaired reads were discarded.

DIAMOND v.0.9.24.125 (blastx –max-target-seqs: 1, –id: 90, –query-cover: 90) (16) was employed to search the cleaned read data sets against NCBI’s Antimicrobial Resistance Reference Gene Database (downloaded 7 November 2018) and the Antibacterial Biocide and Metal Resistance Genes Database v.2.0 (experimentally confirmed resistance genes) (17). The resulting count data were normalized to “relative gene abundance” following the method of reference 18, which accounts for the total number of reads and average read length in each data set and the subject gene length. Furthermore, cleaned data sets were assembled using MEGAHIT v.1.1.3 (–min-count: 2, –min-contig-len: 200, –k-min: 21, –k-max: 127, –k-step: 6).

The relative abundances of ARGs detected in the five samples were 0.4% (S01) to 4.2% (S04) of that detected in the inlet of a wastewater treatment plant (Fig. 1). Antibacterial biocide and metal resistance (BacMet) genes were considerably more abundant, especially in the biofilm exposed to CSI (S05). Surprisingly, this abundance was due to elevated mercury and not copper or silver resistance genes. A range of full-length mercury resistance genes were detected in the assembled data sets. The reason for the high abundance of mercury resistance genes remains unclear.

### Data availability.

All data sets are deposited in ENA (Table 1).

**TABLE 1:** ENA accession numbers and sample indices for the five shotgun metagenomes^*a*^

Sample no.	Sample description	Sample accession no.	Sample barcode	Run accession no.	No. of reads	Assembly accession no.	No. of contigs >1,000 bp	No. of contigs with *N*_50_ >1,000 bp
S01	Cold inlet water	ERS1887712	ATCACGTT	ERR2105748 ERR2105753	46,342,789 44,342,421	ERZ1079234	295,161	3,764
S02	Warm shower water, building without CSI	ERS1887713	CGATGTTT	ERR2163668 ERR2105754	48,595,584 46,497,077	ERZ1079235	300,790	3,756
S03	Warm shower water, building with CSI	ERS1887714	TTAGGCAT	ERR2105750 ERR2105755	53,099,020 50,742,735	ERZ1079236	303,509	3,712
S04	Shower hose biofilm, building without CSI	ERS1887715	TGACCACT	ERR2163669 ERR2105756	57,390,138 53,219,451	ERZ1079237	93,609	14,763
S05	Shower hose biofilm, building with CSI	ERS1887716	ACAGTGGT	ERR2105752 ERR2105757	51,044,779 47,703,704	ERZ1079238	54,579	16,742

a GenBank BioProject number PRJEB22193, and EBI metagenomics (MGnify) study accession number MGYS00001968.
